# Patient complaints differ for male and female obstetrician-gynecologists: an exploration of 20 years of complaints data in Alberta, Canada

**DOI:** 10.1093/intqhc/mzaf091

**Published:** 2025-09-11

**Authors:** Erin A Brennand, Nancy Hernandez-Ceron, Iryna Hurava, Nicole Kain

**Affiliations:** Department of Obstetrics & Gynecology, Cumming School of Medicine, University of Calgary, Calgary, AB, Canada; Department of Community Health Sciences, Cumming School of Medicine, University of Calgary, Calgary, AB, Canada; Research & Evaluation Unit, Analytics, Innovation & Research Department, College of Physicians and Surgeons of Alberta, Edmonton, AB, Canada; Research & Evaluation Unit, Analytics, Innovation & Research Department, College of Physicians and Surgeons of Alberta, Edmonton, AB, Canada; Research & Evaluation Unit, Analytics, Innovation & Research Department, College of Physicians and Surgeons of Alberta, Edmonton, AB, Canada

**Keywords:** Complaint, Disciplinary action, Gender, Regulation, Medical Regulation

## Abstract

**Background:**

Patient complaints are valuable indicators of systemic issues in healthcare, offering opportunities to enhance care quality and safety. Obstetrics and gynecology (OBGYN) is a specialty particularly prone to complaints, reflecting its unique challenges related to patient population, sensitive subject matter, and physician-patient dynamics. The impact of physician sex on patient complaints is not well understood, particularly taking into context the changing demographics of the OBGYN workforce.

**Methods:**

A longitudinal cohort of all obstetrician-gynecologists registered with the College of Physicians & Surgeons of Alberta (CPSA) between 2003 to 2024 linked to the CPSA Complaints database was used. Quantitative analysis included comparisons of mean and proportion, logistic regression, Kaplan-Meier survival, and Cox regression analyses to assess associations between physician characteristics and complaints. Adjusted models accounted for physician sex, birth year, and training location, measured using the Transparency International Corruption Perceptions Index as a proxy for healthcare system similarity to Canada. Content analysis categorized the anonymized complaint narratives provided insights themes of grievances.

**Results:**

The cohort included 449 OBGYN physicians (59.2% female, 40.8% male). Overall, 44.8% of physicians experienced at least one complaint during the study period. Male physicians were more likely to receive complaints than female physicians (52.5% vs. 39.5%), with higher adjusted odds of ethics-related complaints (aOR 2.16, 95% CI 1.12–4.16). Adjusted analyses revealed no significant differences in overall complaint frequency or time to first complaint between male and female OBGYNs. Content analysis highlighted recurring themes of communication, professionalism, and patient-centered care.

**Conclusion:**

While crude analyses suggested sex-based differences in complaint patterns, these associations were attenuated after adjusting for physician age and training location, indicating that the total effect of sex on complaints may be mediated by these factors. However, male physicians remained more likely to receive ethics-related complaints even after adjustment, suggesting a possible direct association. These findings emphasize the need for targeted professional development focused on culturally sensitive communication and patient rapport and underscore the need to consider sex and gender dynamics in OBGYN to promote equity and improve patient care. Future research should explore systemic and structural contributors to complaint patterns across broader geographic and clinical contexts.

## Introduction

Medical complaints provide a unique lens through which to assess the quality and safety of care within healthcare organizations [1, 2]. While clinical outcomes and critical incident reporting systems yield valuable insights, the complaints process stands out as the primary mechanism for capturing the patient voice and experience [3]. Complaints from patients and their families serve as critical indicators of systemic issues within healthcare, particularly in areas such as safety, service quality, poor treatment, and communication [1, 4–7]. Thoughtful analysis of these complaints can foster a culture of safety, offering organizations and individuals opportunities to improve the provision and delivery of care [8].

Recent trends indicate that complaints to regulatory bodies are on the rise, reflecting an expanding pool of data that can be leveraged for research into health system performance and safety [4]. However, complaints research faces challenges due to variations in how complaints are handled and categorized across different health systems and countries [1]. Despite these limitations, emerging research reveals recurring themes and trends, underscoring the importance of sharing findings to identify and implement broad-reaching solutions to patient-identified issues in quality and safety.

Epidemiological studies suggest that surgical specialties are at higher risk of complaints and malpractice lawsuits compared to other fields [9], and female patients are more likely to file complaints than their male counterparts [10, 11]. Notably, research in obstetrics and gynecology (OBGYN) has shown that it is the most complained-about specialty in some contexts, such as in Mexico [10]. There is a compelling rationale for treating OBGYN as a distinct category, as obstetrical care does not fit neatly into the traditional dichotomy of “medicine” versus “surgery.” This distinction poses challenges in defining the role of OBGYN practitioners as either primarily physicians or surgeons. Furthermore, the patient population for the discipline is predominantly biologically female and/or individuals who identify as women, introducing unique sex- and gender-specific considerations into research and evaluation efforts. For instance, while studies indicate that female patients are more likely to lodge complaints, OBGYN lacks a substantial cisgender male patient population to serve as a reference group for comparison.

The sensitive subject matter of reproduction, sexuality, and childbirth further positions this patient population as at-risk and vulnerable. This highlights the importance of investigating complaints within OBGYN, particularly physician-related factors associated with the nature, frequency, and resolution of these complaints. Such research is essential to developing strategies that enhance safety and quality in reproductive healthcare.

In recent years, disparities in clinical outcomes based on physician sex have gained increasing attention, with studies identifying differences in mortality rates, readmissions, and complications between patients treated by male versus female physicians. Within Canada, the proportion of female obstetrician-gynecologists has risen steadily, achieving gender parity in 2012 [12]. Today, over 60% of obstetrician-gynecologists in Canada are female, and it is estimated that more than 80% of current OBGYN trainees are women [13]. Alongside this shift in gender demographics, other physician characteristics, such as the location and recency of medical training, have also evolved over the last two to three decades.

While prior research has shown that patients often prefer female OBGYNs [14], and separate literature confirms that male physicians across all specialties are more likely to be the subject of regulatory complaints [15], there remains a notable research gap at the intersection of these two findings. Specifically, there is a lack of studies examining whether male obstetrician-gynecologists receive a higher number or different types of patient complaints compared to their female counterparts. This gap limits our understanding of how physician sex may influence patient experience and regulatory oversight in a specialty that uniquely blends surgical risk, intimate care, and gendered dynamics.

Given these changes and knowledge gaps, our research group investigated whether physician sex is associated with patient complaints in OBGYN, considering additional physician factors representing experience.

## Methods

### Study design

We conducted a longitudinal analysis of all OBGYN physicians registered with the College of Physicians & Surgeons of Alberta (CPSA) over a 20-year period (November 2004–October 2024).

### Study setting

In Canada, the Canada Health Act enforces five fundamental principles of healthcare, but the responsibility for delivering healthcare rests with individual provinces and territories. These jurisdictions manage healthcare through their own insurance plans, regulatory bodies, and health authority structures [16]. Alberta, located in western Canada and home to approximately 10% of the national population, has a unique healthcare delivery system.

Alberta’s physician regulatory body, the CPSA, operates under the province’s Health Professions Act, which grants self-regulation privileges to physicians [17]. Additionally, Alberta Health Services (AHS)—Canada’s first pan-provincial regional health authority—was established over 15 years ago through the amalgamation of multiple regional health entities [18]. This centralized model enables consistent access to healthcare information, programs, and treatments across Alberta, covering hospitals, emergency rooms, urgent care centers, certain long-term care facilities, and some ambulatory care centers.

In contrast, many Alberta physicians operate independently in private office settings, billing the government on a fee-for-service basis with minimal to no interaction with AHS. While both AHS and CPSA address patient complaints, only CPSA provides a comprehensive dataset, as it handles complaints related to physicians regardless of the care setting. The Research & Evaluation Unit (REVU) within the Analytics, Innovation & Research Department at CPSA evaluates existing programs, supports the development of new ones, and conducts innovative regulatory research to enhance public trust in CPSA-regulated physicians, promote evidence-informed medical regulation, and improve patient care.

### Data source and study cohort

This study utilized the CPSA complaints database, which includes detailed information about public submissions, categorizing them by nature and coding their outcomes post-evaluation. The study cohort comprised all Alberta physicians with active CPSA registration in the general or conditional register specializing in Obstetrics & Gynecology for at least one year between November 2004 and October 2024, inclusive of people practicing subspecialties (*n* = 47) such as Gynecologic Oncology, Reproductive Endocrinology & Infertility, and Maternal-Fetal Medicine.

Physician sex, self-reported at the time of CPSA registration as either male or female, was the primary exposure variable. Additional variables to account for social and cultural factors included birth year and country of undergraduate medical training, which was converted to the Transparency International Corruption Perceptions Index (CPI), a measurement of governance and social inequality used as a proxy for similarity to Canadian healthcare and society [19]; the CPI has previously been shown to be associated with regulatory outcomes in Alberta in unpublished analyses done by REVU.

When a complaint is received by the CPSA, it is initially reviewed and summarized by staff within the Professional Conduct Department. Intake and Resolution Officers, who are trained through structured education, job shadowing, and supervised feedback, use these summaries are then used to classify each complaint into one or more of seven standardized categories based on the complainant’s perspective: (i) Practice Management, (ii) Medical Reporting, (iii) Third Party Issues, (iv) Ethics, (v) Quality of Care, (vi) Systemic Issues and (vii) Unclassified (Supplementary Table 1). Each complaint is subsequently reviewed by a senior director to ensure consistency and quality in coding. A single complaint could involve multiple categories, with outcomes potentially differing between them. For instance, a single complaint could include issues of self-promotion (Ethics) and record accuracy (Medical Reporting); one complaint nature could be upheld and resolved through a mandatory educational activity for the physician, while the other nature could be dismissed.

## Analysis

All analyses were conducted to compare complaint outcomes between male and female obstetrician-gynecologists (OBGYN). The primary study outcome was the occurrence of a CPSA complaint against an OBGYN physician during this period. Secondary outcomes included: (i) the nature(s) of the complaint(s); (ii) frequency of complaints, and (iii) timing of complaints relative to the physician’s year of registration for independent clinical activity in Alberta.

Descriptive statistics were calculated and stratified by physician sex. Bivariate comparisons were calculated, with differences in proportions, means, and medians reported with 95% confidence intervals.

Adjusted models included physician birth year and CPI score of the country where undergraduate medical training was obtained. Models included interaction terms for physician sex with both covariates; model fits were evaluated using the Akaike Information Criterion (AIC). Logistic regression was used for the binary primary outcome of a CPSA complaint between 2004 and 2024, and comparison of complaint natures. Kaplan-Meier and Cox Proportional hazards models were used to examine the timing of a physician’s first complaint, with the first year of registration in Alberta as the starting point. Physicians with complaints during their training period in Alberta were excluded to prevent negative time values. Those with interrupted registrations (e.g. temporary relocation) were censored until their practice resumed.

Summaries of case details from physician complaints between 2004 and 2024 were anonymized. Using NVivo 12 software, complaints were systematically coded and grouped into subcategories within each primary complaint category for content analysis using the CPSA’s established subdivions [20]. For example, the Ethics category was further subdivided into codes such as boundary violations, informed consent, confidentiality breaches, and unprofessional behavior. Exemplar quotes were generated for content categories to provide context as to the nature of these complaint types.

This study was approved by the Conjoint Health Ethics Research Board at the University of Calgary (REB #22-0448). The study adhered to the Strengthening the Reporting of Observational studies in Epidemiology guidelines for cohort studies [21], ensuring rigor and transparency in reporting.

## Results

Between November 1, 2004, and October 31, 2024, there were a total of 449 unique Obstetrician-Gynecologists in the cohort. Of these, 266 self-reported being female (59.2%) and 183 reported male (40.8%). Female OBGYNs reported a more recent year of birth, lower corruption scores of the country of undergraduate medical training, and higher proportions of Canadian medical training; however, the number of years on the CPSA registry was comparable (Table 1).

**Table 1.** Cohort characteristics by female and male sex

Characteristic	All (*N* = 449)	Female (*N* = 266)	Male (*N* = 183)	Difference (95% CI)
Year of birth^a^	1969.7 (15.1)	1976.3 (10.3)	1960.0 (15.8)	16.3 years (13.7–18.9)
Canadian medical school	75.5% (*n* = 339)	87.2% (*n* = 232)	58.5% (*n* = 107)	28.7% (20.1%–37.4%)
Cumulative years in CPSA registry during the observation window^a^	10.5 (6.4)	10.1 (6.50)	11.2, (6.10)	−1.1 years, (−2.3–0.05)
Transparency International Corruption Perceptions Index (CPI) score^a^	69 (15.8)	71.8 (12.9)	64.9, (18.5)	7.0 points (3.9–10.1)

a Mean and standard deviation.

During the cohort window, there were 564 complaints related to Obstetrics & Gynecology which implicated 201 different physicians (44.8% percent of the cohort). Of the Obstetrician-Gynecologists who had experienced complaints, roughly half (46.8%, *n* = 94/201) had only one complaint against them, while the other half (53.2%, *n* = 107/201) faced more than one complaint. Compared by sex, 39.5% of female and 52.5% of male OBGYNs experienced at least one patient complaint; 18.0% of female and 32.2% of male OBGYNs experienced two or more complaints. The median time to first complaint was 11.1 years (95% CI 8.6—13.2) for the coverall cohort; with male OBGYNs having a mean timing of 8.5 years and female OBGYNs experiencing median timing of 12.0 years (Table 2).

**Table 2.** Crude and adjusted estimates of differences in outcomes

Outcome	All (*N* = 449)	Female^a^ (N = 266)	Male (*N* = 183)	Crude estimates^a^ (95% confidence interval)	Adjusted estimates^a^
Proportion with any complaint	44.8% (*n* = 201)	39.5% (*n* = 105)	52.5% (*n* = 96)	OR 1.69, (1.16–2.48)	1.62 (0.95–2.75)
Proportion with ***2 or more*** complaint(s)	23.8% (*n* = 107)	18.0% (*n* = 48)	32.2% (*n* = 59)	OR 2.16 (1.39–3.36)	1.55 (0.87–2.75)
Years from registration to first complaint^b^	11.1 (8.6–13.2)	12.0 (10.7–13.8)	8.5 (6.8–13.4)	HR 1.33 (1.01–1.76)	1.18 (0.86–1.62)
Nature of complaint					
Ethics	14.7% (*n* = 66)	9.0% (*n* = 24)	23.0% (*n* = 42)	OR 3.00 (1.75–5.17)	2.16 (1.12–4.16)
Medical Reporting	8.7% (*n* = 39)	9.0% (*n* = 24)	8.2% (*n* = 15)	OR 0.90 (0.46–1.77)	0.52 (0.23–1.17)
Practice Management	22.9% (*n* = 103)	19.5% (*n* = 52)	27.9% (*n* = 51)	OR 1.59 (1.02–2.48)	1.01 (0.56–1.8)
Quality of Care	35.0% (*n* = 157)	30.1% (*n* = 80)	42.1% (*n* = 77)	OR 1.69 (1.14–2.50)	1.44 (0.85–2.44)
Systemic, Third Party, Unclassified	13.6% (*n* = 61)	10.5% (*n* = 28)	18.0% (*n* = 33)	OR 1.87 (1.09–3.22)	1.90 (1.00–3.64)

HR = hazard ratio, cox proportional model; OR = odds ratio, logistic regression.

a Female physician sex is the referent group.

b Median and confidence interval.

Altogether, 564 unique complaints encompassed 881 distinct complaint natures; the majority of complaints (57.8%, *n* = 326/564) pertained to a single complaint nature. The most prevalent category of complaint pertained to the *Quality of Care* (*n* = 402); followed by *Practice Management* (*n* = 221); *Ethics* (*n* = 123); *Unclassified* (*n* = 73); *Medical Reporting* (*n* = 55); *Systemic* (*n* = 5); and *Third Party* (*n* = 2). Crude differences also existed within nature groups of complaints by physician sex, specifically in the domains of Ethics, Practice Management and Quality of Care (Table 2).

Adjusted models included covariates of year of birth and CPI, as well as length of time on the CPSA register during the twenty-year observational window. Effect modification terms were included for all covariates by sex; effect modification was shown not to exist and AIC indicated improved model fit without the interaction terms resulting in their removal from final models.

**Figure 1.**
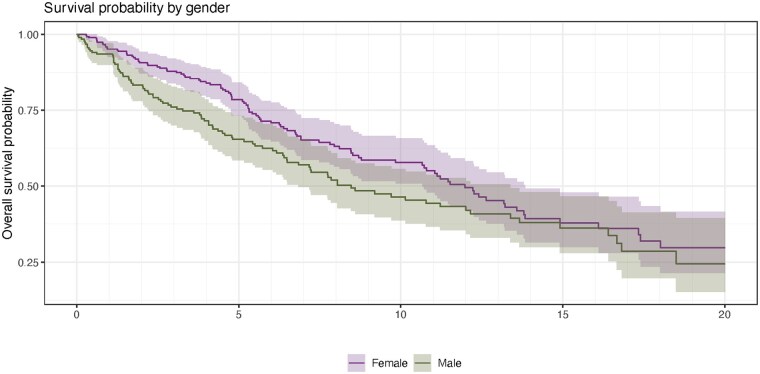
Probability of remaining free of a college complaint by physician sex over 20 years

The adjusted analyses revealed null associations between physician sex and occurrence of a complaint, number of complaints, and time to complaint (Table 2) In adjusted analyses male sex remained associated with increased risk of an Ethics complaint (aOR 2.16, 95% CI 1.12–4.16). Cox Proportional Hazards survival analysis demonstrated that male physicians experienced their first complaint earlier in their registration than females (crude HR 1.33, 95% CI 1.01–1.76, Kaplan Meier Figure 1); use of adjusted models resulted in a null Hazard Ratio (aHR 1.18, 95% CI 0.86–1.62).

Content analysis from the complaint files revealed details about complaints, including boundary violations, confidentiality breaches, informed consent concerns, record accuracy, communication issues, and diagnostic and treatment problems. Salient quotations from the narrative case files are shown in Table 3, highlighting the types of complaints, complaint natures, and emotions conveyed within the textual data and providing a succinct and meaningful representation of the dataset.

**Table 3.** Exemplar quotes from nature groupings, and most common subdivisions of each nature, of OBGYN patient complaints 2004–2024

Nature group—ethics	
Boundary violation—other	*“[Name] was referred to a doctor for cyst removal. The doctor was not professional due to comments she had made about [Name] weight making statements such as you won’t fit in an MRI”.* *“Doctor was rough with internal exam, doctor slid hands up under [Name] two shirts and bra without [Name] permission, doctor ran his fingers along her inner thigh”.*
Confidentiality	*“Saw a doctor for fertility treatments, doctor was rude and belligerent, shouting about appointments and test results. Doctor made staff cry with his attitude”* [complainant expressed concern that these comments were made loudly enough to be overheard by others in the office, raising confidentiality issues]. *“[Name] found a doctor’s communication to be quite rude. Also she feels that a doctor breached confidentiality when sharing the C-Section info to the NICU staff in front of other new moms.”*
Informed consent	*“[Name] had a baby by C section. While on the delivery table after receiving a lot of drugs and an epidural, the doctor asked her if she wanted her tubes tied, [Name] wasn’t paying attention really, but responded “yes”. The doctor got her signature and proceeded to tie her tubes. [Name] feels the doctor should not be obtaining consent under these conditions as she wants more kids.”* *“A doctor sterilized [Name] without her consent.”*

Nature group—medical reporting	
Record accuracy	*“A doctor gave a diagnosis of cancer before one was confirmed, scared the family by making the situation more urgent than necessary.”*

Nature group—practice management	
Communication—­explanation	*“[Name] was referred to gynecologist for bleeding. A doctor refused to examine her and referred her for a mammogram. [Name] was refused because she was under 40. A doctor did not answer [Name] questions and was in the room for only 3 minutes”.* *[Name] asked why a doctor did not do a C-section or provide proper explanation of what happened that caused the loss of her baby.”*
Communication—attitude	*“A doctor did not follow up with pregnancy properly—first baby was born big because of gestational diabetes and the second baby died after 4 days because gestational diabetes was not identified and treated, doctor also did not call mother after the loss of her baby”.* *“[Name] had surgery for HPV, 1st surgery went well, and second one left her in pain and part of her labia falling off. Tried to seek medical attention but everyone referred her back to the initial doctors. [Name] went back and they are not helping her with botched surgery, pain management and recovery”.*
Availability	*“A doctor abandoned [Name] after delivery by not transferring her care and assuring proper follow-up, did not tell other staff at hospital or his clinic that he was leaving town.”* *“No notification given to [Name] that the doctor closed down her clinic. Just a note on the door when [Name] showed up for her appt.”*

Nature group—quality of care	
Diagnosis—delayed	*“[Name] had a tubal ligation and woke up with pain. After several years of persistent pain and seeing different doctors that could not diagnose her problem, it was discovered that the left ovary had been clamped and had flipped itself because of it, the clamps were reset, and her pain was almost gone immediately.”* *“[Name] presented symptoms to the doc and he determined it to be non-obese version of polycystic ovarian syndrome. When told to come back in a month, she could not get in for 3-4 months. When told to come back again in two weeks, again she had a long wait. Doctor wanted to operate and if necessary remove the uterus and ovary even though she would not have consented to this. She ended up with cancer.”*
Diagnosis—incorrect	*“A doctor misdiagnosed the type of ovarian cancer and prescribed the wrong chemo. If [Name] hadn’t gone to Mayo Clinic, [Name] could have died in 6 months instead of the possible 2 to 4 years.”* *“Incorrect diagnosis of cancer was given to patient post surgery. Prescribing of anti-depressants.”*
Follow-up—other	*“Poor follow-up with ultrasound, baby lost fluid and died of undeveloped lungs (also kidneys did not grow).”* *“[Name] had two biopsies which were lost by the doctor’s office and no verbal consultation was given regarding the risks. She ended up with hematoma and a bleed behind her uterus. She had surgery to repair the rectal perforation and uterine perforation, had the colostomy reversed and had a bowel obstruction related to the injury.”*
Treatment-procedural	*“[Name] felt her care was negligent and so poorly managed particularly by “delaying treatment” that it caused a hysterectomy she may not have needed.”* *[Name] had a risky pregnancy. Ob/gyn ignored blood problem/extra fluid in body. [Name] had a c-section and a second surgery because of c-section. Patient nearly died—has many scars and is on lots of meds now.”* *“Nonchalant attitude & carelessness in procedure that led to complications causing [Name] to have the problem corrected by another surgery.”* *“A doctor performed a painful and hurried exam, without concern for patients’ wellbeing.”* *“[Name] went in for a routine hysterectomy. She ended up having three surgeries in total due to issues such as a blockage in her intestines from a mass of infection. The first surgery took 7.5 hours, the second surgery 9 hours. She now suffers from short bowel syndrome and does weekly blood work and maintain vitamins and minerals, sometime having to go to ER for magnesium by IV.”*

## Discussion

### Statement of principal findings

Our study found that in crude analyses, male Obstetrician-­Gynecologists in Alberta were more likely to receive a complaint to the province’s medical regulatory body and earlier time to first complaint. However, adjusted analyses attenuated the associations and made them statistically insignificant, suggesting that older age and higher proportions of physicians who trained in countries with transparency scores different than Canada’s may be mediating factors. However, male sex remained associated with increased risk of an Ethics complaint in adjusted analyses suggesting a direct association.

### Interpretation within the context of the wider literature

Previous research has shown that female physicians often adopt more patient-centered communication styles, which may lead to longer visits [22, 23]. Additionally, over 80% of patients report preferring female OBGYNs [24]. However, female patients may also hold different expectations of female physicians, which can be challenging to meet [14]. Other studies have highlighted gendered differences in patient feedback, with female patients more likely to report feeling “disrespected” in their online reviews of male physicians. At the same time, female physicians tend to receive more criticism regarding their “soft skills” [14]. Within OBGYN specifically, on-line reviews of female OBGYNs often reveal a broader range of patient emotions compared to reviews of male OBGYNs, with a higher likelihood of negative sentiments [25]. These findings collectively underscore the complex gender dynamics between female patients and their reproductive care providers.

Further research has demonstrated that male OBGYNs in the United States are more likely to face disciplinary sanctions from State Medical Boards, with public reviews of sanctioned male physicians tending to be lengthier than those of sanctioned female physicians. This suggests patients may have “more to say” about male providers [25]. Our findings align with this research when male sex is examined independently of other factors, such as age and country of medical training. However, our adjusted analysis indicates that the observed differences in outcomes are more nuanced. Specifically, male physicians are often older and more likely to have trained outside of Canada (or countries with comparable transparency indices), which may account for some of these disparities.

These findings also resonate with broader patterns observed outside of OBGYN. Studies across various medical specialties have shown that patients treated by female physicians often experience better clinical outcomes, including lower mortality rates—differences attributed to more guideline adherent practice styles and greater use of patient-centered care [26, 27]. Furthermore, large-scale studies have found that male physicians, across specialties, are more likely to receive medicolegal actions, even after adjusting for factors such as practice volume and clinical field.^13^ Moreover, analyses of patient complaints in general medical contexts reveal that many arise not from technical failings but from perceived breakdowns in communication, disrespectful interactions, or lack of rapport—issues that transcend specialty and are deeply influenced by provider communication style and cultural competence [1]. Previous work from Australia has demonstrated that graduates trained outside of the country are at increased risk of complaints and adverse disciplinary findings [28], which fits with the interpretation of our study’s adjusted findings which suggest location of training is a mediating factor. It is possible that physicians trained outside of Canada or other high-transparency health systems may face a steeper learning curve in adapting to expectations around patient autonomy, shared decision-making, and relational norms, which may, regardless of specialty, increase their risk of complaints.

Taken together, these broader trends suggest that the patterns we observed in OBGYN, a discipline which focuses specifically on female reproductive health, likely reflect wider systemic issues related to communication, training background, and evolving patient expectations across healthcare. The insights from this OBGYN research may therefore inform more general strategies to improve patient-provider relationships and reduce complaint rates across the medical profession.

### Strengths and limitations

This study has several potential limitations that should be considered when interpreting the findings. First, the analysis was confined to a single province, resulting in a relatively small sample size of 449 physicians over a 20-year period. This limited sample size may reduce statistical power, decrease precision, and contribute to wider confidence intervals, which could impact the robustness of the conclusions. Additionally, Alberta’s unique healthcare environment—characterized by a publicly funded, single-payer model and a high proportion of fee-for-service OBGYNs—may limit the generalizability of the findings to jurisdictions with differing economic and healthcare delivery systems. Research has shown that reimbursement structures can significantly influence patient care and physician behavior, potentially creating contexts that differ substantially from those observed in Alberta [29]. Although our models adjusted for important physician-related factors such as age and country of training, residual confounding by unmeasured variables remains a possibility. Factors such as differences in subspecialization, practice settings, or patient case mix could potentially influence outcomes and were not accounted for in this analysis. The narrative data used in this study were derived from notes created by CPSA intake staff during the initial handling of each complaint. As such, the data reflect staff summaries rather than direct transcripts or full accounts provided by complainants. This limits the depth of the material for qualitative analysis, as not all details or nuances shared by the complainant may have been captured. Consequently, the analysis could only support the identification of broad thematic patterns. While the CPSA’s system of categorizing complaints enables structured analysis, it is important to recognize that it is based on staff interpretations rather than unfiltered patient-reported content. Moreover, the reliance on regulatory data, while valuable, may not fully capture the complexity of patient-physician dynamics or the broader spectrum of professional performance issues.

### Implications for policy, practice and research

Future research could address these limitations by expanding the scope of the study to include additional provinces and larger sample sizes. Pooling data across multiple studies using meta-analytic techniques would provide a more comprehensive, pan-Canadian perspective on the relationship between physician sex in OBGYN and regulatory outcomes. Such an approach would enhance the generalizability of findings and allow for exploration of regional variations within Canada’s healthcare system. Replicating prior research on gender differences in online physician reviews would also be valuable, particularly as patients increasingly share their experiences and concerns in public forums. These narratives may influence how future patients interpret provider competence, communication style, and trustworthiness—often before any direct interaction occurs. As a result, online reviews may shape expectations, reinforce gender-based assumptions, and influence the dynamics of subsequent clinical encounters, with important implications for the therapeutic relationship and perceived quality of care. Additionally, comparative analyses in jurisdictions with different healthcare and reimbursement models, such as those in the United States or Europe, could also provide valuable insights into how systemic factors influence the observed sex and gender dynamics.

Our findings underscore the need for targeted support and educational resources for male OBGYNs, particularly those who are older and who have trained in systems that are fundamentally different than Canada. This could include training programs and workshops focused on culturally sensitive communication and patient-centered care, as well as efforts to address complex or emotionally charged patient interaction scenarios, which commonly lead to patient complaints. Emphasizing qualities such as empathy, clear communication, and shared decision-making—regardless of physician sex—can help mitigate complaints. Studies show that patients prioritize these attributes over gender when selecting providers [30]. For male OBGYNs, these skills can help bridge gaps in perceived care quality and address potential biases or misalignments in patient expectations. Additionally, mentorship programs could play a critical role in supporting internationally trained physicians, helping them navigate the nuances of the Canadian health care system, including it’s cultural, legal, and professional expectations, as well as reinforcing communication strategies that align with the expectations and needs of Canadian patients.

It is also important to consider the broader implications of the increasing feminization of the OBGYN workforce. While this trend aligns with patient preferences for gender-concordant care and may enhance patient satisfaction, it carries the risk of reinforcing and exacerbating existing biases against male providers [31]. Such biases could contribute to a perception that male OBGYNs are less suited for the specialty, potentially leading to their marginalization within the field. This shift may also inadvertently reduce diversity of thought and approach, which are critical for innovation and comprehensive care in any medical specialty.

To mitigate these risks, efforts should be made to foster a culture of inclusivity and equity within OBGYN, ensuring that all providers, regardless of gender, are supported and valued. This includes addressing potential biases in medical education, recruitment, and professional development, as well as fostering patient education initiatives that highlight the importance of provider competencies and communication skills over gender. Future research should also explore how changing gender demographics within OBGYN impact both patient care and physician experiences, providing insights that can guide policies to promote equity and excellence in the specialty.

## Conclusions

In summary, while the higher rate of complaints against male OBGYNs may partly reflect systemic issues such as age-related factors, international training backgrounds, and gender biases, our findings point to actionable opportunities. Interventions that promote culturally sensitive communication, enhance support for internationally trained physicians, and address gendered expectations in care may help to improve patient-provider relationships and reduce complaint risk. Future research should evaluate the effectiveness of such strategies in promoting equity and professionalism within OBGYN and beyond.

## Supplementary Material

mzaf091_Supplementary_Data

## Data Availability

The data underlying this article may be shared on reasonable request. Data requests should be directed to the College of Physicians and Surgeons of Alberta.
