# Supplementary material for: Patient complaints differ for male and female obstetrician-gynecologists: an exploration of 20 years of complaints data in Alberta, Canada
Source: Int J Qual Health Care. 2025 Sep 11;37(4):mzaf091. doi: 10.1093/intqhc/mzaf091 (PMC12532309; doi:10.1093/intqhc/mzaf091)
Supplement: mzaf091_Supplementary_Data [file mzaf091_supplementary_data.docx]

**Supplementary Table 1**. Definitions and complaint natures within each CPSA Complaint Category

| Category | Description | Included Complaint Natures |
| --- | --- | --- |
| Quality of Care | Concerns related to clinical decision-making, treatment, follow-up, referrals, and diagnostic accuracy. | Assessment, Diagnosis, Treatment  Diagnosis – Incorrect  Diagnosis – Delayed  Follow-up – Other  Follow-up of Significant Test Results  Referral/Consultations  Treatment – Counseling  Treatment – Prescribing  Treatment – Procedural  Treatment – Procedures |
| Practice Management | Issues related to physician availability and the administration or environment of the medical office. | Availability  Communication – Attitude  Communication – Explanation  Financial  Office – Environment  Office – Scheduling  Office – Staff Relations |
| Medical Reporting | Complaints concerning the handling, accuracy, or timeliness of medical reports or health information. | Record Accuracy  Report Completion  Release of Records |
| Ethics | Violations of ethical standards, including confidentiality, informed consent, boundaries, and professional conduct. | Advertising/Self-promotion  Boundary Violation – Financial  Boundary Violation – Other  Boundary Violation – Sexual  Confidentiality  Informed Consent  Professional Behavior  Regulatory Compliance  Sexual Misconduct |
| Third Party | Issues related to third-party medical assessments and documentation, often for insurers or WCB. | Independent Medical Examinations and related interactions |
| Systemic | Concerns involving coordination of care, interdisciplinary care, or system-level challenges. | Continuity of Care  Interdisciplinary  Access – Technology |
| Unclassified | Complaints that do not clearly fit into the above categories or are too general to categorize. | All other complaint types not captured in the defined categories |
